# LARGE-SCALE POPULATION-LEVEL DATA FOR STUDYING GRANDPARENTING IN THE UNITED STATES

**DOI:** 10.1093/geroni/igad104.0855

**Published:** 2023-12-21

**Authors:** Sarah Flood

**Affiliations:** University of Minnesota, Minneapolis, Minnesota, United States

## Abstract

The questions we can answer about grandparenting in the United States are shaped by the availability of data. This presentation will review previous research on trends and sociodemographic variation in grandparenting in the United States based on large-scale population-level data from IPUMS (www.ipums.org). It will highlight the various ways that researchers have measured families that include grandparents (e.g., three-generation families, grandfamilies). It will also discuss the limitations of these data, specifically the focus on coresidence, for studying grandparents. The presentation will then review research on grandparents using the American Time Use Survey, the only data in IPUMS that enables analyses of grandparents time spent with both their resident and non-resident grandchildren. These analyses will focus on the leisure activities that grandparents share with their grandchildren and how this varies by living arrangements and sociodemographic characteristics.

